# Insight into a Nitrogen-Doping Mechanism in a Hard-Carbon-Microsphere Anode Material for the Long-Term Cycling of Potassium-Ion Batteries

**DOI:** 10.3390/ma15124249

**Published:** 2022-06-15

**Authors:** Changdong Chen, Kai Zhao, Ming La, Chenghao Yang

**Affiliations:** 1 School of Enviornment and Energy, South China University of Technology, Guangzhou 510006, China; lmccd5613@163.com; 2 College of Chemistry and Environmental Engineering, Pingdingshan University, Pingdingshan 467000, China; 3 College of Information Engineering, Pingdingshan University, Pingdingshan 467000, China; zhaokai@pdsu.edu.cn

**Keywords:** potassium-ion batteries, hard-carbon microspheres, nitrogen-doping, energy storage mechanisms

## Abstract

To investigate the alternatives to lithium-ion batteries, potassium-ion batteries have attracted considerable interest due to the cost-efficiency of potassium resources and the relatively lower standard redox potential of K^+^/K. Among various alternative anode materials, hard carbon has the advantages of extensive resources, low cost, and environmental protection. In the present study, we synthesize a nitrogen-doping hard-carbon-microsphere (N-SHC) material as an anode for potassium-ion batteries. N-SHC delivers a high reversible capacity of 248 mAh g^−1^ and a promoted rate performance (93 mAh g^−1^ at 2 A g^−1^). Additionally, the nitrogen-doping N-SHC material also exhibits superior cycling long-term stability, where the N-SHC electrode maintains a high reversible capacity at 200 mAh g^−1^ with a capacity retention of 81% after 600 cycles. DFT calculations assess the change in K ions’ absorption energy and diffusion barriers at different N-doping effects. Compared with an original hard-carbon material, pyridinic-N and pyrrolic-N defects introduced by N-doping display a positive effect on both K ions’ absorption and diffusion.

## 1. Introduction

In recent years, energy storage devices represented by lithium-ion batteries (LIBs) have been widely used in various fields to accelerate the goal of carbon neutrality [1,2,3]. However, limited lithium resources cannot satisfy the burgeoning demand of LIBs [4]. Thus, it is particularly important to develop new energy storage devices. As one of the alkali metal-ions, potassium-ion is rich in resources and has similar physicochemical properties to lithium-ions; furthermore, potassium-ion batteries (PIBs) also deliver a reaction mechanism similar to LIBs, which belongs to the “rocking chair batteries” [5,6]. In comparison to sodium (−2.71 V vs. standard hydrogen electrode), the standard potential of K^+^/K (−2.93 V vs. SHE) is relatively close to that of Li^+^/Li (−3.04 V vs. SHE), which suggests that PIBs have the potential of working in a broad voltage window and thus delivering a high energy density [7,8]. Additionally, in PIB electrolytes, due to the weaker Lewis acidity and smaller de-solvation activation energy of K ions than those of Na and Li, K ions show a smaller Stokes radius and interfacial reaction resistance in electrolytes, which display a more rapid conductivity and better interfacial reaction kinetics of K ions [9]. Due to those advantages, PIBs have attracted considerable interest as an alternative candidate for energy storage systems (ESSs).

To date, the design of anode materials is the key to developing PIBs. In consideration of the similar development of anode materials in LIBs, several anode materials have attracted much attention, mainly including carbon-based materials [10], metal oxides [11,12], and alloy-type materials [13,14]. Although potassium-ions intercalated into graphite carbon layers can form a thermodynamically stable intercalation compound, its larger radius (0.138 nm) hinders the rapid intercalation behavior between the carbon layers and shows unsatisfactory specific capacity [15,16]. Recently, hard carbon with extended carbon-layer spacing has received increasing attention from researchers and has been demonstrated as an anode for PIBs [17]. Compared to graphite, the arrangement of graphite-like microcrystallines in hard carbon is disordered, and there are only a few regularly stacked microcrystalline regions. Hard carbon mainly consists of a sp2-hydridized poly-hexagonal carbon-ring structure [18]. The contained intrinsic defects in hard carbon induce the dislocation and deformation of carbon-ring structures, leading to a long-range disordering structure with the large and irregular d-spacing [19]. Those expanded graphitic lattices of hard carbons could be more suitable for K ions’ (de)intercalation. At the same time, hard carbon has the advantages of extensive resources, low cost, and being environmentally friendly, which attracts much interest concerning its role as a promising anode for PIBs [20,21]. However, pure hard carbon also suffers from a low specific capacity and poor rate capability.

To solve this limitation, extensive ideas have been presented; heteroatom-doping, in particular, is the usual modified strategy [22,23]. Nitrogen-doping is one of the most popular doping strategies, which can not only provide additional active defects in hard carbon, but also improve conductivity by tuning the intrinsic electronic state [24]. Urea is used as a dopant, and Deng et al. [25] designed an N-doped three-dimensional biomass-derived porous carbon from bagasse. The experimental results show that the introduction of nitrogen not only promotes the change in morphology, but also accelerates the transport of potassium-ions and electrons. Even following 400 cycles at a current density of 200 mA g^−1^, the reversible capacity of the modified derived carbon can maintain 100.4 mAh g^−1^ without significant capacity decay. In addition, Zhou et al. [26] synthesized an N-doped carbon hollow turbostratic tube by a amidation reaction and heating treatment process. Benefiting from the doping modification of the N atom, the discharge capacity of the carbon hollow turbostratic tube reaches 397 mAh g^−1^ at 0.1 A g^−1^, and the capacity of 212 mAh g^−1^ can be maintained, even when the current density is increased to 2 A g^−1^. Furthermore, when the scan rate of cyclic voltammetry (CV) is 0.6 mV s^−1^, the electrode has 71% capacitance contribution, corresponding to 46.3% of the battery, implying that capacitively controlled processes dominate at high rates. Wang et al. [27] synthesized the edge-enrich N-doped graphitic carbon via pyrolyzing carbon nitride for the PIB anode. Owing to the high N-doping level, the edge-enrich N-doped graphitic carbon could deliver a high capacity of 266 mAh g^−1^ and a remarkable rate performance of 228.9 mAh g^−1^ at 2 A g^−1^. Moreover, an ultra-long lifespan is displayed in edge-enriched N-doped graphitic carbon, which maintains 188.9 mAh g^−1^ even after 2200 cycles.

The above reports all confirm that N-doping can significantly improve the electrochemical performance of hard carbon, but there are many types of N-doping configurations, which are generally divided into pyridinic-N, pyrrolic-N, and graphitic-N, and their respective effects are rarely reported [28,29]. In a previous report, it was suggested that the graphitic-N site in hard carbon could enhance the electronic conductivity of carbon materials, and pyrrolic-N and graphitic-N could be capable of providing more active sites for K ions’ adsorption [30]. However, a systematic evaluation of the effects of K-ion diffusion after N-doping is still lacking. In this work, N-doped hard-carbon microspheres were obtained by using glucose and melamine as the precursors, combined with the hydrothermal reaction and high-temperature heat-treatment process. The differences in the electrochemical performance before and after doping are compared in detail. Furthermore, we employ the Density Functional Theory (DFT) calculation to assess the effect on K^+^ absorption and, subsequently, K-ion diffusion of different N-doping effects, which confirms the positive role of pyridinic-N and pyrrolic-N in enhancing the electrochemical properties of hard-carbon microspheres.

## 2. Experimental Section

**Material synthesis and characterization:** the hard-carbon microspheres were synthesized via the hydrothermal reaction. A total of 6.4 g glucose (99% purity, Macklin) was dissolved in 45 mL of deionized water and the solution was poured into an autoclave. After being heated at 200 °C for 5 h, the black powders were collected by filtration and drying at 80 °C. To obtain the N-SHC, the obtained powder was mixed with melamine (99% purity, Macklin), and subsequently annealed at 900 °C for 5 h in an argon atmosphere. For the original SHC sample, the black powders collected by filtration were heat treated directly at 900 °C for 5 h in the argon atmosphere without melamine. The structural features of the obtained materials were determined by powder X-ray diffraction (XRD) recorded by CuKα radiation (λ = 1.5418 Å) in the scan range of 10–70°. Field-emission scanning electron microscopy (SEM, Hitachi S-4800) and transmission electron microscopy (TEM, JEOL-JEM-2100) were employed to characterize the morphologies and microstructures. The surface chemical states were analyzed using X-ray photoelectron spectroscope (XPS, PHI500 Versaprobe-II).

**Electrochemical measurements:** the electrochemical properties were evaluated in the 2032 coin-type cells. The working electrodes were prepared by coating the pulp suspension, which consisted of 80% active material, 10% super P, and 10% carboxymethyl cellulose (CMC) on copper (Cu) foil. After drying at 80 °C in a vacuum oven overnight, the copper foil was cut into 13 mm electrodes and assembled in an Ar gas-filled glovebox. For the assemblage of coin-type cells, the employed electrolyte was 0.8 M KPF6 in ethylene carbonate and propylene carbonate (EC:PC = 1:1), glass fiber (Whatman) was used as the separator, and metallic potassium served as a counter electrode. The galvanostatic charge-discharge tests were conducted in the battery measurement system (Land, CT2001), and the cyclic voltammogram (CV) and electrochemical impedance spectroscopy (EIS) in the frequency range of 0.01–100 kHz were performed in the electrochemical work station (CHI660E).

**Computational Methods**: The modeling and simulation were based on the spin-polarized density functional theory (DFT), which employed the Vienna Ab initio Simulation Package (VASP). The simulation adopted the Perdew–Burke–Ernzerhof test for the exchange-correlation potential and projector-augmented wave (PAW) with a setting cut-off energy of 400 eV. The Brillouin zone was modeled as a structure of 3×3×2. The diffusion barriers of K ions between the adjacent carbon layer were calculated by using the CI-NEB [31].

## 3. Results and Discussion

**Figure 1:**
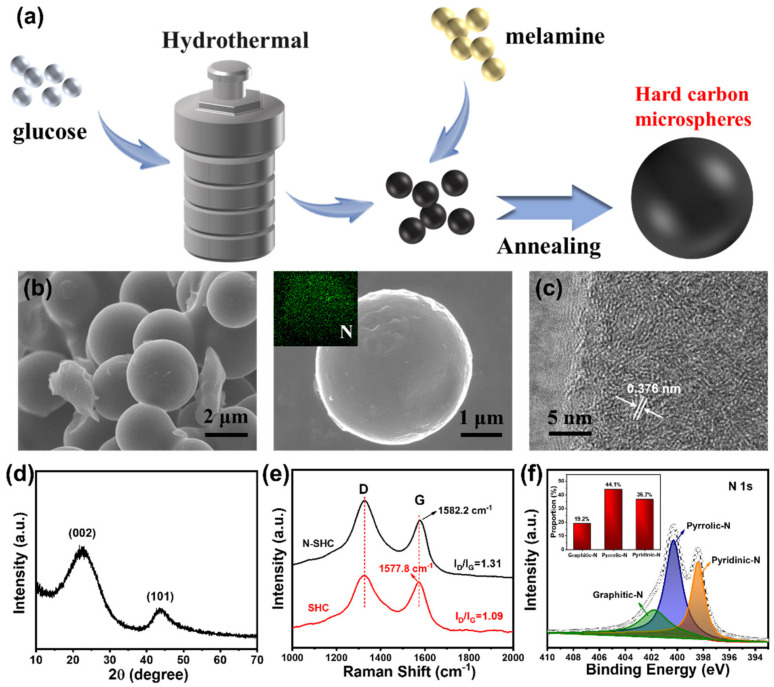
The (**a**) schematic of the synthesis process, (**b**) SEM images and EDS element mapping of as-prepared N-SHC, (**c**) TEM image, (**d**) XRD pattern, (**e**) Raman spectra and (**f**) high-resolution XPS spectra of nitrogen in N-SHC.

The morphologies of as-prepared N-SHC and SHC samples were investigated by SEM and TEM. As show in Figure 1b, the obtained N-SHC sample consists of uniform spherical hard-carbon particles with diameters of around 3 μm. Additionally, the energy-dispersive X-ray spectroscopy (EDS) mapping (inserted in Figure 1b) of N-SHC displays a homogeneous distribution of the N element on the hard-carbon sphere. The TEM was employed to observe the microstructure further. As depicted in Figure 1c, the average interlayer distance of N-SHC is 0.376 nm, which is greater than the original SHC sample (0.349 nm in Figure S1). The expanded interlayer space of N-SHC should contribute to the nitrogen groups introduced by N-doping, which provides a buffer for the structural change during the insertion–extraction process of K^+^ ions.

Figure 1d represents the XRD patterns of N-SHC and SHC. Broad diffraction peaks appear at around 25° in both N-SHC and SHC samples, which can be indexed on the (002) plane [32]. Additionally, the weak diffraction peak at about 43° suggests the relatively low degree of graphitization. Compared to SHC (24.4°), the (002) peak of N-SHC shifts to a lower 2θ angle (23.6°), indicating the enlarged interspaces of N-SHC, which is in accordance with the analysis of TEM. Additionally, Figure 1e displays the Raman spectra of N-SHC and SHC. The peaks centered at around 1330 and 1580 cm^−1^ are ascribed to the disorder/defect-induced D band and in-plane vibrational G band, respectively [33]. The intensity ratios (I_D_/I_G_) of D and G peaks have been established to assess the degree of graphitization and the amount of disorder defects [34]. It can be observed that the I_D_/I_G_ value of SHC is 1.09 and the I_D_/I_G_ ratio of N-SHC is calculated to be 1.31, revealing the higher degree of disorder defects induced by N-doping. Moreover, there appears to be a slight blue shift in the G band in N-SHC (1585.2 cm^−1^), which results from increased carrier concentration via N-doping [35]. Figure 1f represents the high-resolution X-ray photoelectron spectroscopy (XPS) of the N element in N-SHC. This can be deconvoluted as the peaks in the N 1s spectrum of N-SHC: the peak at 401.8 eV is indexed to graphitic-N; peaks at 400.8 and 398.6 eV correspond to pyrrolic-N and pyridinic-N, respectively [36]. Accordingly, the nitrogen concentration in the N-SHC sample was determined as 6.3% by the XPS analyses. Moreover, the proportion of various N-defects were calculated via convolution and the results are inserted in Figure 1f. Previous research has mentioned the high chemical activity of pyrrolic-N and pyridinic-N. These high relative concentrations of pyrrolic-N and pyridinic-N in N-SHC could lead to better chemical properties for K^+^ ions’ storage.

**Figure 2:**
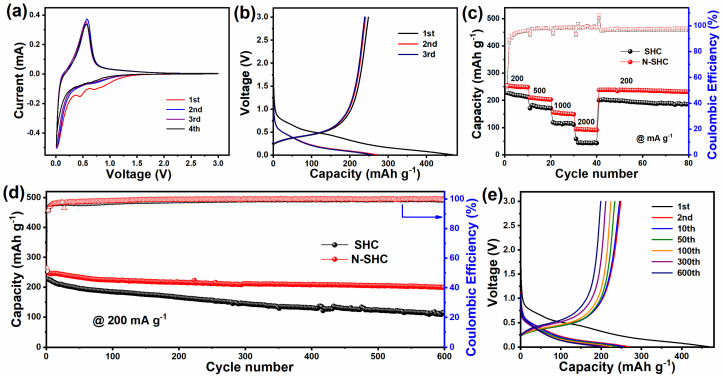
(**a**) CV curves and (**b**) charge-discharge profiles of the N-SHC electrode. Compared (**c**) rate performance and (**d**) long-term cycle properties at 200 mA g^−1^ for N-SHC and SHC electrodes. (**e**) Corresponding charge-discharge profiles of N-SHC at 200 mA g^−1^ after various cycles.

The K-ion storage properties of as-prepared N-SHC and SHC were investigated by the galvanostatic test, using potassium metal as the counter electrode. Figure 2a demonstrates the typical cyclic voltammetry (CV) curve for the N-SHC material at the potential window of 0.01–3.0 V. There were two cathodic peaks centered at ≈0.5 and 0.75 V that could be observed in the first cycle, but disappeared in the following cycles. The cathodic peaks that disappeared could probably be attributed to the formation of a solid electrolyte interface (SEI) layer. The anodic peak at about 0.55 V and corresponding sharp cathodic peak at 0.12 V should be ascribed to the de-intercalation and intercalation processes of K^+^ ions forming into carbon, respectively. The overlap at CV curves between second and third cycles revealed a stable cycling capability. However, an obvious difference was noticed when comparing the first cathodic process with the second one, which suggested a relatively low initial coulombic efficiency (ICE) of N-SHC. The ICE of the anode material is defined as the ratio of charge capacity and discharge capacity in the first cycle. This can also be confirmed by the charge/discharge profiles. Figure 2b shows the potassiation/depotassiation profiles of the initial three cycles for N-SHC at 1 C (where 1 C is defined as 200 mA g^−1^ in this work). The first potassiation capacity of N-SHC is 466 mAh g^−1^ with the depotassiation capacity of 248 mAh g^−1^, which reveals an ICE of 53%. The ICE of the N-SHC electrode is similar to SHC (51%), whose initial discharge/charge capacities are 446/228 mAh g^−1^ (Figure S2). The promotion of the reversible capacity of N-SHC (248 mAh g^−1^) compared to SHC (228 mAh g^−1^) can be noted. This high capacity of N-SHC may be attributed to the enhanced adsorption effect, which is triggered by N-doping in N-SHC sample. In the first cycle of both the N-SHC and SHC samples, three quasi-plateau regions and a high-potential sloping region can be observed, and the plateau at ≈0.8 V versus K^+^/K disappears in the following potassiation process. This irreversible reaction may be related to the formation of the solid electrolyte interface (SEI) and should be responsible for the low ICE [37].

The rate performances of N-SHC and SHC samples were evaluated at various current densities. It was interesting to note that the rate properties of N-SHC were improved, where the N-SHC electrode delivered charge capacities of 251, 206, 152, and 93 mAh g^−^^1^ at the increased current densities of 200, 500, 1000, and 2000 mA g^−^^1^, respectively, as shown in Figure 2c. Compared to the SHC sample, both samples displayed a similar rate capacity retention at a low current rate of 500 mAh g^−1^. However, as the current densities increased to 1000 and 2000 mA g^−1^, the capacity of SHC declined more rapidly than the N-SHC electrode. At the rates of 1000 and 2000 mA g^−1^, N-SHC presented 152 and 93 mAh g^−1^, respectively, while the reversible charge capacities of pristine SHC were 116 and 43 mAh g^−1^ at the same current densities. When the current density returned to 200 mA g^−1^, the reversible charge capacity of N-SHC was recovered to 245 mAh g^−1^ following the high-current-rate test, while SHC returned to 200 mAh g^−1^. This indicates the stabler structure of the N-SHC material compared to SHC. The better rate performance of N-SHC may be due to N-doping leading to a lower diffusion barrier for K^+^-ion transfer.

The long-term cycling properties of N-SHC and SHC are evaluated at 200 mA g^−1^. As shown in Figure 2d, the SHC electrode delivers an initial charge capacity of 228 mAh g^−1^. Additionally, the reversible capacity of SHC rapidly decayed to 178 mAh g^−1^ after 100 cycles at 200 mA g^−1^, corresponding to a capacity retention of 78%. Compared to SHC, the N-SHC shows a better cycle performance. After cycling at 200 mA g^−1^ for 100 cycles, N-SHC presented a higher capacity of 224 mAh g^−1^ with a capacity retention of 90%. When cycled further, at 600 cycles, the superiority of long-term cycling properties of N-SHC were featured more obviously. After 600 cycles at 200 mA g^−1^, the capacity retention of SHC was no more than 50%. Surprisingly, the N-SHC electrode maintained a high reversible capacity at 200 mAh g^−1^ after 600 cycles, which displayed a superior capacity retention of 81%. Furthermore, Figure 2e and Figure S3 exhibit the charge/discharge profiles after different cycles of N-SHC and SHC samples, respectively. It can be observed that, compared to the SHC electrode, N-SHC shows a slight voltage polarization. This capacity fading and voltage polarization may be a result of the pulverization of SHC particles, where the spherical particle of the N-doping N-SHC sample reveals better structural stability. The structural stability of the N-SHC sample also can be verified by electrochemical impedance spectroscopy (EIS). Figure S4 represents the Nyquist plots of N-SHC and SHC electrodes after various cycles. The Nyquist plots consist of a semicircle and a straight line. The interception of the semicircle corresponds to the contact ohmage, the semicircle in the high-frequency regions is relevant to the charge transfer resistance, and the straight line in the low-frequency region is associated with the Warburg impedance, which reflect ion transportation in the electrode [38]. It can be fitting that the initial charge transfer resistance (R_ct_) of the SHC electrode is 4868 Ω, and continues decline to 1797 Ω after 100 cycles, which should be attributed to the activization of the electrode. Then, R_ct_ begins to increase and, after 300 cycles, the R_ct_ of SHC increases to 3921 Ω, while N-SHC delivers the R_ct_ of 2982, 684, and 1139 Ω at the original rate and after 100 and 300 cycles, respectively. For comparison, the lower-charge transfer resistance and minor change in the N-SHC electrode after cycling indicates the improved conductivity and better cyclability of N-doping N-SHC.

**Figure 3:**
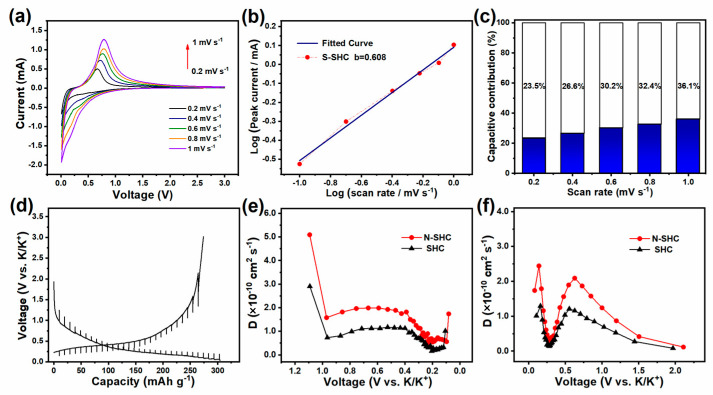
(**a**) CV curves of N-SHC at scan rates from 0.2 to 1 mV s^−1^, corresponding (**b**) relationship between log (peak current) versus log (scan rate) and (**c**) calculated normalized percentages of capacitive capacities’ contributions at various scan rates. (**d**) GITT profiles and diffusion coefficients in (**e**) discharge and (**f**) charge processes.

To obtain further insight into the dynamic characteristic of the potassium-ion-storage behavior involved in the N-SHC electrode, we employed CV analysis at various scan rates from 0.1 to 1 mV s^−1^. Figure 3a shows the CV test result of N-SHC. Additionally, the electrochemical-reaction behavior was assessed by the relationship of the peak-current (i) response with the scan rate (v), according to Equations (1) and (2):

(1) $$i=a\cdot v^{b}$$

(2) $$\log\left(i\right)=b\cdot \log\left(v\right)+\log\left(a\right)$$

where i is the peak current, v is the scan rate, and the calculated a and b are adjustable parameters [39]. In particular, the storage behavior can be reflected by parameter b; when b is close to 0.5, the K^+^ ion’s storage behavior is dominated by the diffusion process; and when b approaches 1.0, the capacitive process predominates. The b-value could be determined by the slope of the plotted log(i)−log(v) curve [40]. In this case, as displayed in Figure 3b and Figure S5, the b-values for N-SHC and SHC are calculated as 0.608 and 0.527, respectively, indicating that the diffusion process predominates in the K^+^-ion storage behavior. Furthermore, the capacitive contribution fractions could be distinguished based on Equation (3):

(3) i=k1·v12+ k2·v

where k_1_∙v^1/2^ is related to the diffusion-controlled reaction, k_2_∙v represents a surface-driven capacitive process, and k_1_ and k_2_ are constants [41]. Figure 3c shows the calculated capacitive contribution fractions of the N-SHC electrode at different scan rates, and the capacitive contribution ratio reaches 36.1% at 1 mV s^−1^. Compared to pristine SHC (Figure S5), the surface-driven capacitive behavior of N-SHC is promoted, which should be ascribed to the generation of active defects on the surface induced by N-doping and enhances the adsorption of K^+^ ions.

**Figure 4:**
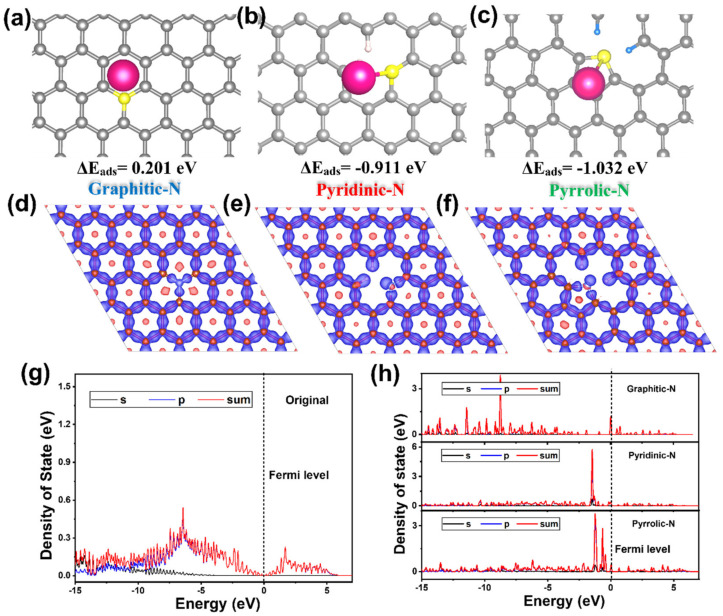
The DFT illustrations of adsorption energy of K ions (set adsorption energy at original hard carbon as 0 eV) and differential charge densities in (**a**,**d**) graphitic-N, (**b**,**e**) pyrindic-N, and (**c**,**f**) pyrrolic-N defects. The density states of (**g**) original hard carbon and (**h**) various defects of N−doping hard−carbon materials.

Furthermore, the galvanostatic intermittent titration technique (GITT) test was employed to evaluate the diffusion ability of K^+^ ions in electrodes. Figure 4d exhibits the GITT profiles with the test condition of a pulse current density of 20 mA g^−1^ for 30 min and 2 h rest intervals. The corresponding ionic diffusion coefficients are determined based on Fick’s second law as the following equation:

(4) $$D=\frac{4}{\pi \cdot \tau }\cdot {\left(\frac{m_{B}\cdot V_{m}}{M_{B}\cdot S}\right)}^{2}\cdot {\left(\frac{\Delta E_{S}}{\Delta E_{\tau }}\right)}^{2}\;\;\;\;\;\;\left(\tau \ll \;L^{2}/D\right)$$

Here, the labels *D*, *m_B_*, *V_M_*, *M_B_*, and *S* correspond to the K^+^-ions diffusion coefficient, the active mass of electrode, molar volume for carbon, molar mass for carbon, and active surface area, respectively [42]. Additionally, the detailed parameters in the GITT test, on which the diffusion coefficient calculations are based, are illustrated in Figure S6, where τ represents the duration of the pulse current and ΔE_τ_ and ΔE_s_ represent the voltage variations during the pulse current and adjacent rest steps. Figure 3e displays the diffusion coefficients of N-SHC and the SHC electrode during the insertion process, where diffusion coefficients first decline until ≈ 0.2 V, and then begin to recover. The corresponding diffusion coefficients during de-potassiation are represented in Figure 3f. It is interesting to note that the diffusion coefficients first decrease, then recover until they attain a voltage value of 0.55 V, and then decline again. Additionally, an increase in the K^+^-ion diffusion coefficient of N-SHC can be observed during both the charge and discharge processes. We attribute this faster K^+^-ion migration in N-SHC to the defect introduced by N-doping, which reduces the diffusion barriers of K^+^ ions.

To interpret the reaction mechanism of K^+^ ions’ storage behavior, the differential charge density and adsorption abilities of different N-doping defects were investigated. Figure 4a–c simulate the K^+^ ions’ adsorption energy for three kinds of N-doping-surface defects on hard carbon (where the adsorption energy on original hard carbon was set as 0 eV). In contrast, the K^+^ ions’ adsorption energies on various introduced N-doping-surface defect sites of graphitic-N, pyrindic-N, and pyrrolic-N were calculated to be 0.201 eV (Figure 4a), −0.911 eV (Figure 4b), and −1.032 eV (Figure 4c), respectively. The DFT calculation exhibited the relatively low adsorption energy of graphitic-N, which indicated a less attractive tendency to K^+^ ions in this kind of N-doping defect. Higher K^+^-ion adsorption energies can be observed at the pyrindic-N and pyrrolic-N active sites, which reveals a stronger driving force and tendency for K^+^ ions to be caught by those defects. This stronger adsorption of surface sites induced by N-doping is beneficial to enhancing the capacitive behavior of hard carbon, especially showing a positive effect on improving the reversible capacity of the N-SHC sample. The distinguishable adsorption energies between the graphitic-N site and pyridinic-N and pyrrolic-N sites could be verified by the differential charge density around N-doping defects. Figure 4d–e show the differential charge densities around graphitic-N, pyridinic-N, and pyrrolic-N sites, respectively. Heterogeneous electron distribution and electron vacancy appear around the pyridinic-N and pyrrolic-N defects, which cause the electron cloud of K^+^ ions to be inclined to be combined with pyridinic-N and pyrrolic-N defects. The electron-rich structure that shows a negative effect on K^+^-ion adsorption at the graphitic-N site should be responsible for the relatively low adsorption energy of graphitic-N [43]. Additionally, the density of states (DOSs) of original hard carbon and different N-doping sites were investigated (Figure 4g,h). It can be observed that, compared to the original one (Figure 4g), the DOSs around the Fermi level increase as we introduce all three kinds of N-doping defects into the original hard carbon. This increases in DOS around the Fermi level suggest the promotion of electronic conductivity and a higher affinity with K^+^ [44].

**Figure 5:**
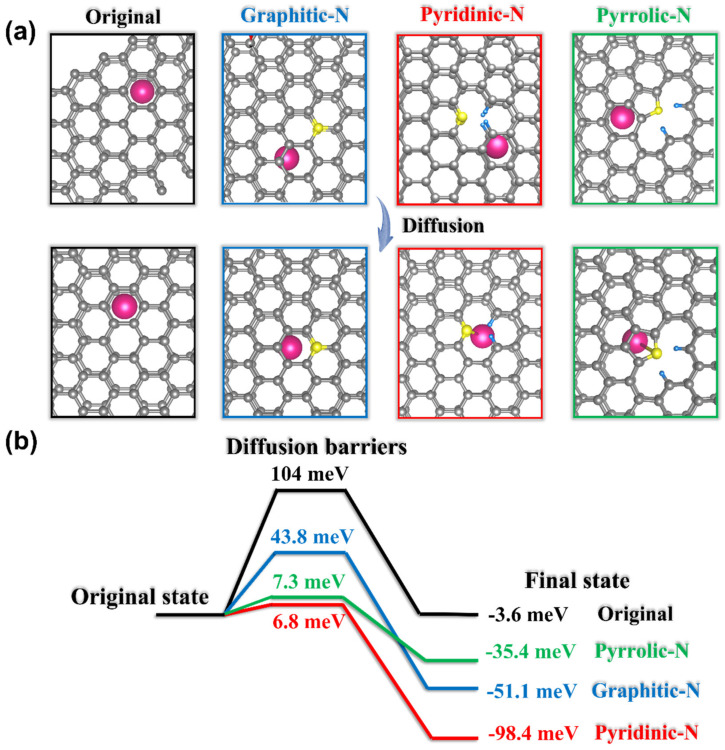
(**a**) DFT illustrations of K-ion diffusion, and (**b**) simulated diffusion barriers and thermodynamics across original hard carbon, graphitic-N, pyrindic-N, and pyrrolic-N defects.

To further solidify the improvement of diffusion kinetics in N-doping hard-carbon materials, we assessed the K^+^ ions’ migration barriers in the situation of K^+^ ions across N-doping defects (Figure 5). Original hard-carbon material displays the largest K^+^-ion migration barrier of 104 meV and lowest diffusion thermodynamics of −3.6 meV, which should be responsible for the sluggish rate capacity of the SHC electrode. In comparison, when the K^+^ ions passed through the hard-carbon structures with graphitic-N, pyridinic-N, and pyrrolic-N defects, the migration barrier of K^+^ ions was simulated to be 43.8, 7.3, and 6.8 meV, respectively. Obvious decreases in the K^+^-ion migration barriers induced by N-doping appeared, particularly in the pyridinic-N and pyrrolic-N defects. This promoted the diffusion of K^+^ ions between adjacent C layers more easily. Additionally, the diffusion thermodynamics across graphitic-N, pyridinic-N, and pyrrolic-N defects were calculated as −51.1, −35.4, and −98.4 meV, which revealed a stronger driving force for K^+^-ion migration. Those simulation results could explain the better rate capacity delivered by N-doping the N-SHC electrode.

## 4. Conclusions

In summary, we synthesized nitrogen-doping hard-carbon microspheres of N-SHC material as an anode for PIBs. In PIBs, N-SHC delivers a high reversible capacity of 248 mAh g^−1^ and a promoted rate performance (251, 206, 152, and 93 mAh g^−1^ at 200, 500, 1000, and 2000 mA g^−1^, respectively). Moreover, nitrogen-doping N-SHC material also exhibits superior cycling long-term stability, where the N-SHC electrode maintains a high reversible capacity at 200 mAh g^−1^ with a capacity retention of 81% after 600 cycles. DFT calculations assessed the change of K ions’ absorption energy and K ions’ diffusion barriers at different N-doping effects. Compared with original hard-carbon material, pyridinic-N and pyrrolic-N defects introduced by N-doping displayed the positive effects of both K-ion absorption and diffusion. Based on the experimental observations and theoretical calculations, the superior electrochemical properties of N-SHC are ascribed to the enhancement of K-ion absorption and migration induced by N-doping.

## Data Availability

The data presented in this study are available upon request from the corresponding author.

## Supplementary Materials

The following supporting information can be downloaded at: https://www.mdpi.com/article/10.3390/ma15124249/s1. Figure S1: (a) SEM and (b) TEM images of SHC; Figure S2: XRD pattern of SHC; Figure S3: (a) The initial charge-discharge profiles of SHC. And (b) Charge-discharge profiles of SHC at various cycles; Figure S4: EIS Nyquist plots of (a) SHC and (b) N-SHC after various cycles; Figure S5: (a) The initial CV curves of SHC at the scan rate of 0.1 mV s^−1^. (b) CV curves of SHC at scan rates from 0.2 to 1 mV s^−1^, corresponding (c) Relationship between log (peak current) versus log (scan rate) and (d) calculated normalized percentages of capacitive capacities contribution at various scan rates; Figure S6: The detailed GITT profiles of N-SHC; Figure S7: The GITT profiles of SHC; Table S1 Comparison of the electrochemical performance of N-doping hard carbons. References [45,46,47,48] are cited in the supplementary materials.
