# Supplementary material for: Insight into a Nitrogen-Doping Mechanism in a Hard-Carbon-Microsphere Anode Material for the Long-Term Cycling of Potassium-Ion Batteries
Source: Materials (Basel). 2022 Jun 15;15(12):4249. doi: 10.3390/ma15124249 (PMC9229776; doi:10.3390/ma15124249)
Supplement: Supplementary file 1 [file materials-15-04249-s001.zip › materials-1741067-supplementary.pdf]

Support Information

Insight to Nitrogen-doping Mechanism in Hard Carbon Microspheres Anode Material for Long-term Cycling Potassium-Ion Batteries

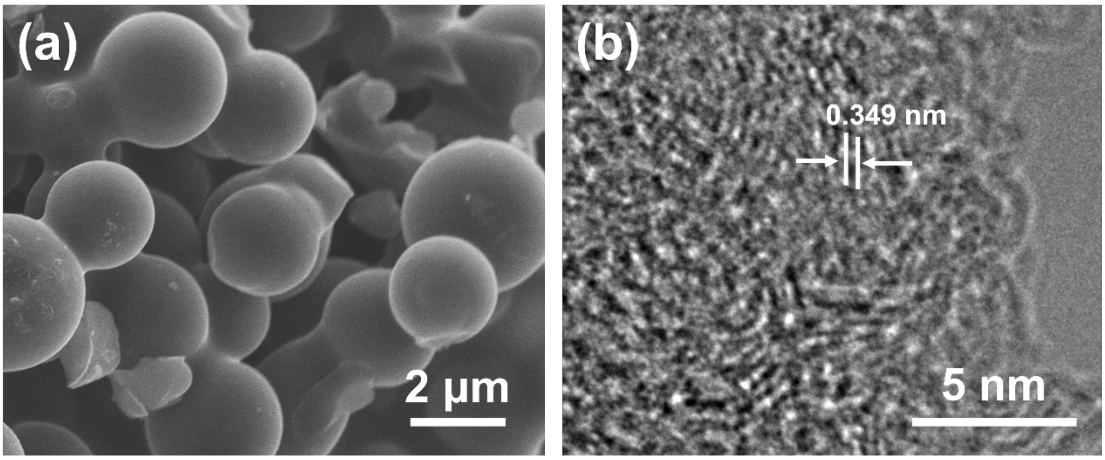

Figure S1. (**a**) SEM and (**b**) TEM images of SHC.

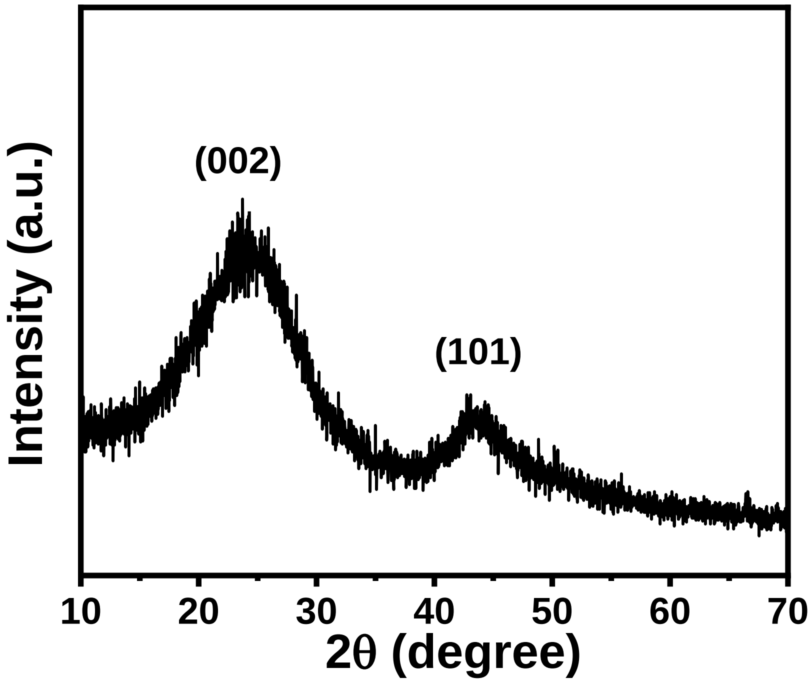

Figure S2. XRD pattern of SHC.

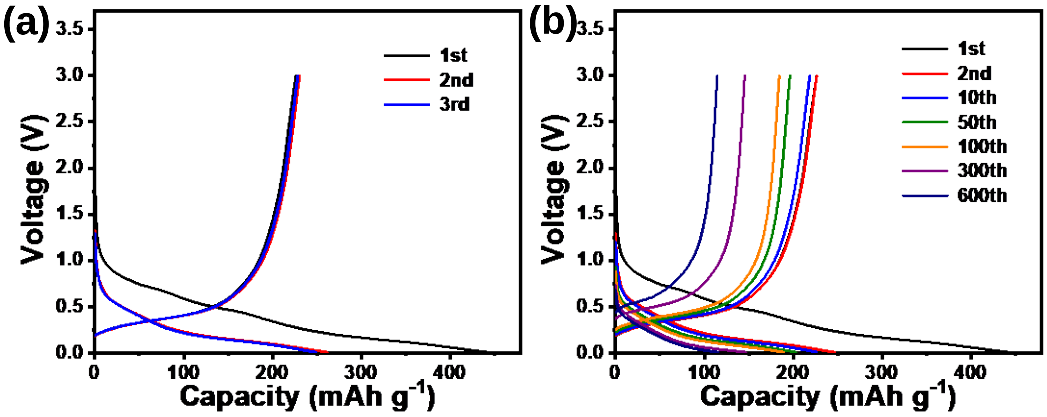

Figure S3. (a) The initial charge-discharge profiles of SHC. And (b) Charge-discharge profiles of SHC at various cycles.

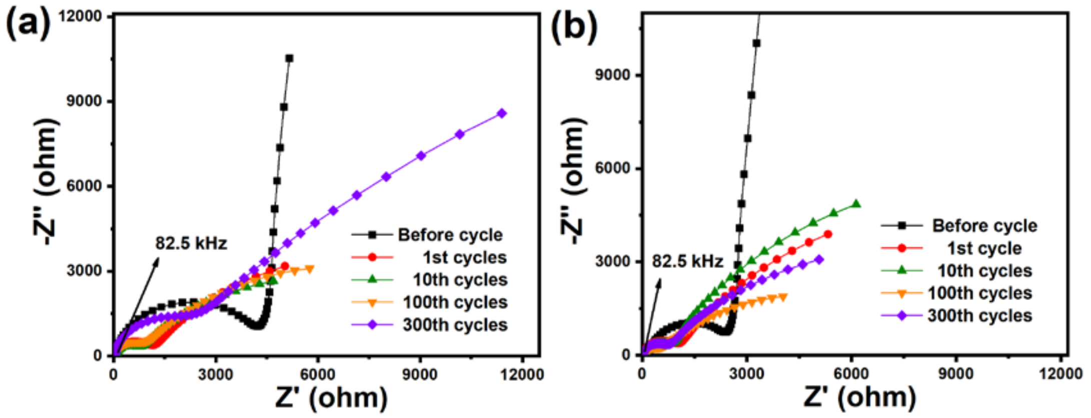

Figure S4. EIS Nyquist plots of (a) SHC and (b) N-SHC after various cycles.

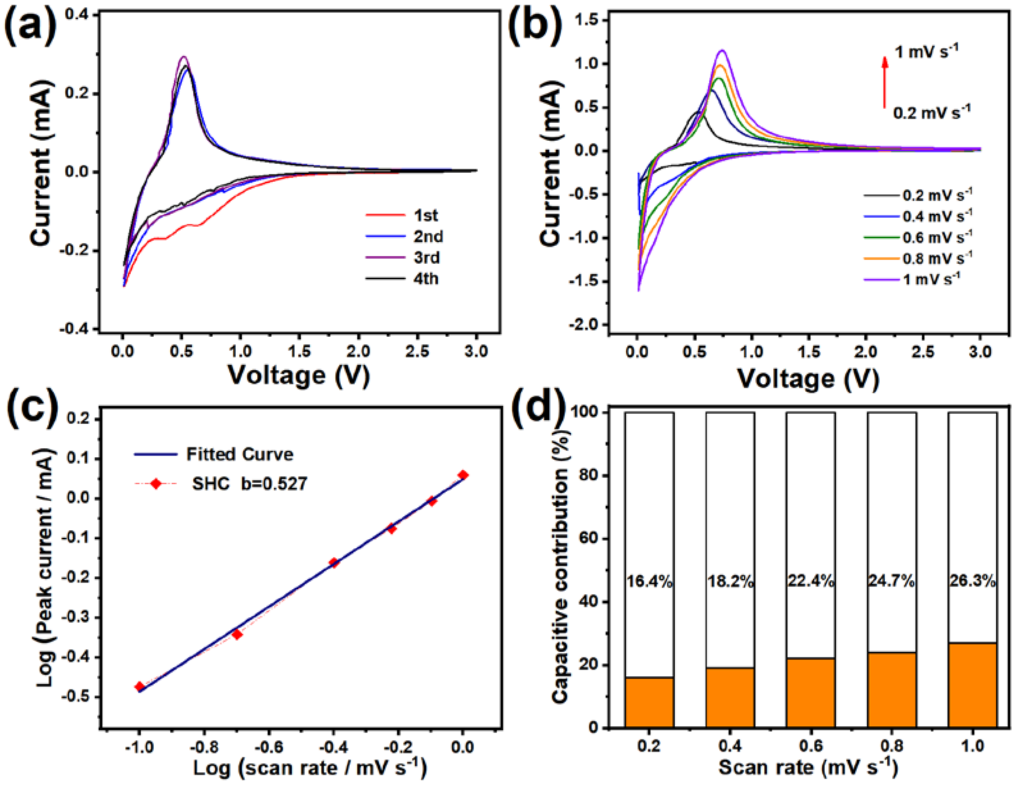

Figure S5. (a) The initial CV curves of SHC at the scan rate of 0.1 mV s^{-1}. (b) CV curves of SHC at scan rates from 0.2 to 1 mV s^{-1}, corresponding (c) Relationship between log (peak current) versus log (scan rate) and (d) calculated normalized percentages of capacitive capacities contribution at various scan rates.

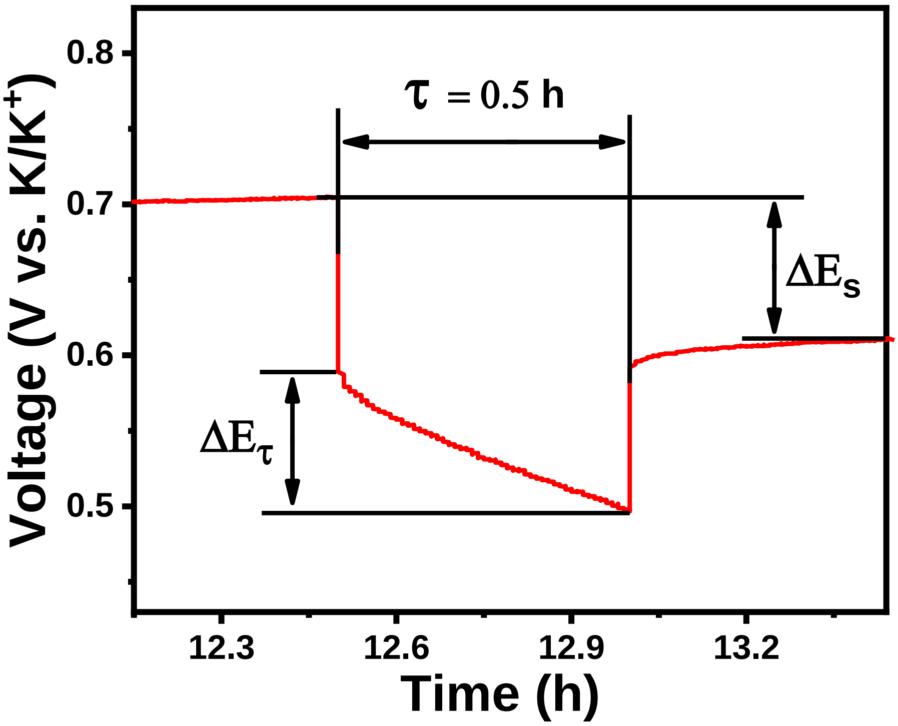

Figure S6. The detailed GITT profiles of N-SHC.

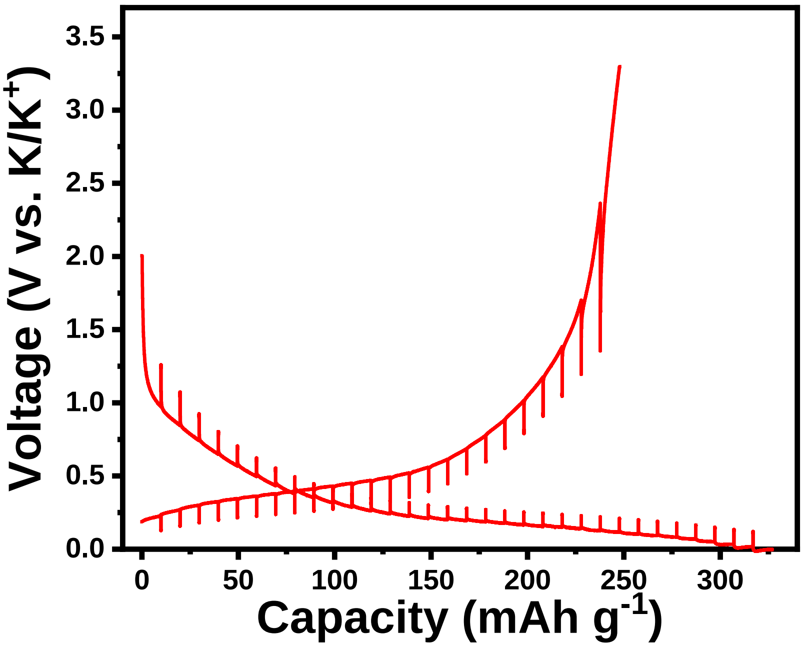

Figure S7. The GITT profiles of SHC.

Table S1 Comparison of the electrochemical performance of N-doping hard carbons

Materials	Doped atom	ICE	Performance	Ref.
TCNs	Nitrogen	24.3%	290.4 mAh/g at 100 mA/g<br>119.7 mAh/g at 2000 mA/g	[1]
DS	Nitrogen	46.9%	168 mAh/g at 50 mA/g<br>49 mAh/g at 1000 mA/g	[2]
MS	Nitrogen	39.9%	123 mAh/g at 50 mA/g<br>30 mAh/g at 1000 mA/g	[2]
SP	Nitrogen	35.7%	139 mAh/g at 50 mA/g<br>43 mAh/g at 1000 mA/g	[2]
SCNNi	Nitrogen	28.1%	142 mAh/g at 100 mA/g	[3]
N-CNF	Nitrogen	52%	193 mAh/g at 50 mA/g<br>106 mAh/g at 1000 mA/g	[4]
SHPNC	Nitrogen	-	273 mAh/g at 50 mA/g<br>78 mAh/g at 5000 mA/g	[5]
NMC	Nitrogen	37.7%	301.9 mAh/g at 50 mA/g<br>133.3 mAh/g at 5000 mA/g	[6]
N-SHC	Nitrogen	53%	251 mAh/g at 200 mA/g<br>93 mAh/g at 1000 mA/g	This work

[1] Zhu L F, Zhang Z, Zhang H, et al. Tunable 2D tremella-derived carbon nanosheets with enhanced pseudocapacitance behavior for ultrafast potassium-ion storage. Science China Technological Sciences, 2021, 64(9): 2047-2056.

[2] Wang X, Zhao J, Yao D, et al. Bio-derived hierarchically porous heteroatoms doped-carbon as anode for high performance potassium-ion batteries. Journal of Electroanalytical Chemistry, 2020, 871: 114272.

[3] Deng Q, Liu H, Zhou Y, et al. N-doped three-dimensional porous carbon materials derived from bagasse biomass as an anode material for K-ion batteries. Journal of Electroanalytical Chemistry, 2021, 899: 115668.

[4] Ma L, Li J, Li Z, et al. Ultra-stable potassium ion storage of nitrogen-doped carbon nanofiber derived from bacterial cellulose. Nanomaterials, 2021, 11(5): 1130.

[5] Luo H, Chen M, Cao J, et al. Cocoon silk-derived, hierarchically porous carbon as anode for highly robust potassium-ion hybrid capacitors. Nano-micro Letters, 2020, 12(1): 1-13.

[6] Qiu Z, Zhao K X, Liu J, et al. Nitrogen-doped mesoporous carbon as an anode material for high performance potassium-ion batteries. Electrochimica Acta, 2020, 340: 135947.
